# A Comparative Analysis of Risk Scoring Systems in Predicting Clinical Outcomes in Upper Gastrointestinal Bleed

**DOI:** 10.7759/cureus.26669

**Published:** 2022-07-08

**Authors:** Abhijnya K Renukaprasad, Srikanth Narayanaswamy, Vinay R

**Affiliations:** 1 Internal Medicine, M S Ramaiah Medical College, Bangalore, IND; 2 Anaesthesia and Critical Care, M S Ramaiah Medical College, Bangalore, IND

**Keywords:** upper gastrointestinal bleed, intensive care unit, hematemesis, gastrointestinal hemorrhage, blood transfusion

## Abstract

Background

Upper gastrointestinal bleed (UGIB) is a life-threatening condition that presents as hematemesis (fresh blood), coffee-ground vomiting, or melena. Multiple scoring systems are developed to predict different clinical outcomes, which are important to managing UGIB and are essential to determining low and high-risk patients. The study aimed to compare the sensitivity and specificity of risk scoring systems and their optimum cut-off values in the assessment of UGIB.

Methods

The prospective cross-sectional study included patients (N = 81) with acute UGIB. Four different proposed scores [Glasgow-Blatchford score (GBS), AIMS65, pre-endoscopic Rockall, and full Rockall scoring system] were used for evaluating patients with UGIB. The optimum cut-off values of these risk scores were used to predict the clinical outcomes.

Results

The AIMS65 score [Area Under the Receiver Operating Characteristic curve (AUROC): 0.91, cut-off: >1, sensitivity: 100%, specificity: 76.62%] and pre-Rockall were similar (AUROC: 0.91, cut-off: >0, sensitivity: 100%, specificity: 93.51%) at predicting mortality. The GBS (cut-off: >9, AUROC: 0.79, sensitivity: 69.23, specificity: 87.50) and AIMS65 scores (cut-off: >0, AUROC: 0.67, sensitivity: 72.31, specificity: 62.5) were good predictors of need for ICU care.

Conclusion

GBS was superior in predicting categorization into high risk and low risk, and endoscopic intervention, blood transfusion, and intensive care unit (ICU) care in UGIB patients. Pre-Rockall score and AIMS65 score were similar in predicting the mortality rate in UGIB.

## Introduction

Upper gastrointestinal bleed (UGIB), defined as bleeding occurring from the gastrointestinal tract, presents as hematemesis (fresh blood), coffee-ground vomiting, or melena (black stools) [1]. The presentation of patients varies widely with an insignificant bleed or may have significant bleed which may lead to death. The estimated number of UGIB is 48-165 per 100,000 adults/year with a mortality rate of 6-10% overall. However, the number varies in different regions of the world [2, 3]. Peptic ulcer disease and gastrointestinal variceal bleeding secondary to portal hypertension are the two leading causes of UGIB [3, 4].

Multiple scoring systems are developed to predict different clinical outcomes in UGIB patients. It is increasingly noticed that early identification of high-risk patients is an essential part of management, as it directly recommends suitable patient care, and also the timing of endoscopy. With multiple risk scoring systems being in place for UGIB, there are ones that can be calculated without the endoscopy as a component [5]. An ideal risk score is one that is easy to calculate, one with high sensitivity for determining outcomes, and can be calculated early during a presentation with UGIB. However, the accuracy and generalisability of these scores and the optimum cut-offs to distinguish low-risk from high-risk patients, remain unclear [2, 6].

Glasgow Blatchford score (GBS), Rockall score (RS), and the AIMS65 score are the typically used risk scoring systems for UGIB [7, 8]. Our study intended to frame optimum threshold values of the scoring systems to assess predetermined composite endpoints, which would help in the quick assessment and risk stratification in patients with UGIB [5]. The study aims to compare the sensitivity and specificity of risk scoring systems and their optimum cut-off values in the assessment of UGIB.

## Materials and methods

The prospective cross-sectional study was conducted in acute UGIB patients attending a tertiary care hospital from October 2018 to July 2020. After Institutional Ethics Committee approval from Ramaiah Medical College (vide letter number EC/PG-27/2018) and written consent, all consecutive patients attending the hospital with acute UGIB (defined by hematemesis, coffee-ground vomiting, or melena, within 7 days of onset) were included [9, 10]. Patients who did not undergo upper gastrointestinal endoscopy or attended the hospital after 7 days of onset of UGIB were excluded. Subjects (N = 81) included were calculated based on the reported prevalence of gastrointestinal bleeding of 4.7 [9], and 7% absolute allowable error at 95% confidence level and 80% power.

Data collection

Patient demographic including clinical history recorded. Physical, systemic, and routine blood investigations were performed. Upper gastrointestinal endoscopy was done in all the UGIB patients, and findings were noted. If any growth is noted, then a biopsy was taken and sent for histopathological analysis.

Scores in UGIB

Four different proposed scores (GBS, AIMS65, pre-endoscopic Rockall, and full Rockall scoring system) were used for evaluating UGIB. GBS includes clinical (history, comorbidities, and systolic blood pressure) along with laboratory variables (blood urea, hemoglobin), which have a score ranging from 0-23 [7, 11, 12]. A detailed description of GBS can be found in Table 1. AIMS65 includes five clinical and laboratory parameters (serum albumin <3g/dL, INR >1.5, altered mental status [Glasgow Coma Scale (GCS) <14/15, stupor, coma] systolic blood pressure <90 mmHg, age >65 years), each corresponding to 1 point [8, 11]. Admission Rockall (pre-endoscopic Rockall) scoring system ranging from 0-7 score has three variables i.e., age (<60 years, 60-79 years or ≥80 years), comorbidities (no major comorbidity, any comorbidity except renal failure, liver failure, and/or disseminated malignancy, renal failure, liver failure, and/or disseminated malignancy) and shock (no shock, tachycardia or hypotension) [11, 13]. Table 2 depicts, that the full Rockall scoring system has a score ranging from 0-11 and has variables including age, comorbidity, shock, diagnosis, and major stigmata of most recent hemorrhage [11, 13].

**Table 1:** Glasgow-Blatchford bleeding score

Admission risk marker	Score component value
Blood urea (mmol/L)	
6.5–8.0	2
8.0–10.0	3
10.0–25	4
> 25	6
Haemoglobin (g/dL) for men	
12.0–12.9	1
10.0–11.9	3
<10.0	6
Haemoglobin (g/dL) for women	
10.0–11.9	1
<10.0	6
Systolic blood pressure (mm Hg)	
100–109	1
90–99	2
<90	3
Other markers	
Pulse ≥ 100/min	1
Melaena	1
Syncope	2
Hepatic disease	2
Cardiac failure	2

**Table 2:** Full Rockall scoring system UGIT: upper gastrointestinal tract, IHD: ischemic heart disease, MW: M-Weiss tear, GI: gastrointestinal, BP: blood pressure

Component score	0	1	2	3
Age	<60	60–79	≥80	
Haemodynamics				
Pulse (bpm)	<100	≥100		
Systolic Blood Pressure (mmHg)	≥100	≥100	<100	
Comorbidities	None		IHD, cardiac failure, other major comorbidities	Renal or liver failure, disseminated malignancy
Diagnosis	MW or no lesion and no stigmata	All other diagnoses	Malignant lesions of UGIT	
Stigmata of haemorrhage	No stigmata or dark spot on ulcer		Blood in UGIT, adherent clot, visible/spurting vessel	

The optimum cut-off values of these risk scoring systems were studied for the following clinical outcomes: requirement of blood transfusion; endoscopic treatment, interventional radiology, or surgery; in-hospital death or duration of in-hospital stay; and mortality.

Statistical analysis

R 4.0.3 (R Foundation for Statistical Computing, Vienna, Austria) was used to analyze the data. QQ plot/Shapiro-Wilk’s test was used to check the normality of variables. Continuous variables are presented as mean ± SD form and categorical variables as a frequency table. GBS Score, AIMS65 score, pre-Rockall, and full Rockall scores were further analyzed using the receiver operating characteristic for determining optimal cut-off points to calculate sensitivity, specificity, positive predictive value (PPV), and negative predictive value (NPV). The comparison of >3 groups was done with the Kruskal-Wallies test. p<0.05 represents the statistical significance.

## Results

Patients (N=81) with UGIB were studied. Table 3 presents the demographic including clinical characteristics of the UGIB patients. Most patients aged 51 to 65 years with male predominance (75%). Ethanol (82.35%) was the common cause of liver disease. The majority (43.2%) of UGIB patients had underlying liver disease (41.98%). The patients having variceal (46.9%) and non-variceal bleed (48.1%) were found to be almost similar. Around 52% of patients required transfusion of blood and 53.1% underwent endoscopic intervention, especially banding (51.16%) followed by sclerotherapy (25.58%). More than three-fourths (80.25%) required ICU care; While only a few patients died from UGIB (4.94%).

**Table 3:** Descriptive statistics for demographic and clinical variables APC: Argon plasma coagulation; yrs: years

Variables	Mean (SD)
Age	55.91 (15.74)
Duration of hospitalization	5.05 (3.00)
Age group	Frequency
21-35 yrs	10 (12.35%)
36-50 yrs	18 (22.22%)
51-65 yrs	34 (42.98%)
66-80 yrs	16 (19.75%)
>80 yrs	3 (3.70%)
Gender	
Female	20 (24.69%)
Male	61 (75.31%)
Comorbidity	
Ischemic heart disease	15 (18.52%)
Chronic kidney disease	7 (8.6%)
Malignancy	3 (3.7%)
Liver disease	34 (41.98%)
Aetiology of liver disease	
Ethanol	28 (82.35%)
Nonalcoholic steatohepatitis	4 (11.76%)
Hepatitis B infection	2 (5.88%)
Habits	
Smoking	15 (18.52%)
Alcohol	32 (39.51%)
Aetiology of UGIB	
Variceal	38 (46.9%)
Non-variceal	39 (48.1%)
Occult	4 (4.93%)
Endoscopic intervention	
APC	1 (2.33%)
Banding	22 (51.16%)
Banding + Sclerotherapy	1 (2.33%)
Haemospray	2 (4.65%)
Sclerotherapy + clipping	5 (11.63%)
Stenting	12 (27.91%)
Blood transfusion	
No	39 (48.1%)
Yes	42 (51.9%)
Requirement of Intensive care	
No	16 (19.75%)
Yes	65 (80.25%)
Mortality	
No	76 (93.80%)
Yes	5 (6.20%)

No significant difference was observed in the distribution of AIMS65, GBS, pre-Rockall, and full-Rockall over the duration of hospitalization (p>0.05; Table 4). Even though the GBS score was higher, it did not show any association with the duration of hospital stay. Although patients with a lower score (<8) stayed in the hospital for a longer duration, it is for other reasons such as electrolyte imbalances and their other comorbidities, i.e., chronic kidney disease on maintenance hemodialysis, myocardial infarction, and sepsis.

**Table 4:** Comparison of Glasgow-Blatchford Score, AIMS65 score, pre-Rockall, and full Rockall scores with hospital stay GBS: Glasgow-Blatchford bleeding score

Variables	Hospital stay (Mean [SD])			P Value
	1-5 days	6-10 days	11-15 days	
AIMS65	0.96 (0.84)	0.94 (1.20)	2 (1.41)	0.104
GBS	9.16 (4.19)	9.94 (3.85)	8.71 (5.15)	0.703
Pre-Rockall	2.96 (1.43)	2.71 (2.02)	3.43 (1.40)	0.433
Full Rockall	4.02 (1.68)	3.64 (2.32)	4.43 (1.27)	0.427

By univariate logistic regression, for a unit increase in AIMS65 and pre-Rockall, the odds of patients who died increased by 5.60 (1.87, 31.82) times and 2.26 (1.15, 5.38) times significantly more than the odds of patients who have survived (Table 5). With the unit increase in GBS, the odds of patients who required endoscopic intervention were 1.12 (1.01, 1.26) times significantly more than the odds of patients who did not require intervention (Table 5). With the unit increase in AIMS65 and GBS, the odds of patients who required ICU care were 2.17 (1.12, 4.93) times and 1.31 (1.13, 1.55) times significantly more than the odds of patients who did not require ICU care, respectively (Table 5). AIMS65 (p = 0.01) and pre-Rockall (p = 0.02) were better at predicting mortality (Table 5). The GBS score was better at predicting requirement for endoscopic intervention (p = 0.049), requirement for ICU (p < 0.001), and blood transfusion (p = 0.00) when compared with other scoring systems (Table 5). AIMS65 was also better at predicting the need for ICU (p = 0.03), however, quite low compared to GBS.

**Table 5:** Comparing of Glasgow-Blatchford Score, AIMS65 score, pre-Rockall, and full Rockall scores to predict the clinical outcomes GBS: Glasgow-Blatchford bleeding score; AUC: area under the curve

Variable	Coefficient	Odds ratio (95% CI)	P value	AUC (95% CI)
Mortality				
AIMS65	1.72	5.60 (1.87, 31.82)	0.01*	0.91 (0.82,1)
GBS	1.87	6.46 (0.91, 115.92)	0.05	0.98 (0.96, 1)
Pre-Rockall	0.81	2.26 (1.15, 5.38)	0.02*	0.91 (0.81, 1)
Full Rockall	0.52	1.68 (0.99, 2.98)	0.051	0.89 (0.80, 1)
Endoscopic intervention required				
AIMS65	0.24	1.28 (0.82, 2.03)	0.28	0.59 (0.47, 0.71)
GBS	0.11	1.12 (1.01, 1.26)	0.049*	0.62 (0.50, 0.62)
Pre-Rockall	0.06	1.07 (0.80, 1.43)	0.65	0.51 (0.38, 0.63)
Full Rockall	0.13	1.14 (0.89, 1.48)	0.31	0.53 (0.41, 0.65)
ICU required				
AIMS65	0.78	2.17 (1.12, 4.93)	0.03*	0.67 (0.53, 0.82)
GBS	0.27	1.31 (1.13, 1.55)	<0.001*	0.79 (0.67, 0.92)
Pre-rockall	0.24	1.27 (0.89, 1.85)	0.19	0.60 (0.46, 0.74)
Full rockall	0.19	1.21 (0.89, 1.71)	0.23	0.60 (0.46, 0.74)
Blood transfusion required				
AIMS65			0.32	0.55
GBS			0.00*	0.71
Pre-Rockall			0.21	0.59
Full Rockall			0.22	0.56

AIMS65 classification was 96.30% accurate to predict mortality with a cut-off value of >2. GBS classification was 61.73% accurate to predict endoscopic intervention with a cut-off value of >9. GBS classification was 72.84% accurate to predict ICU care with a cut-off value of >10 (Table 6).

**Table 6:** Comparison of sensitivity, specificity, PPV and NPV along with cut-off value of Glasgow-Blatchford Score, AIMS65 score, pre-Rockall, and full Rockall scores to predict mortality, endoscopic intervention, ICU care, and blood transfusion GBS: Glasgow-Blatchford bleeding score; PPV: Positive predictive value; NPV: Negative predictive value

Variables	Cut off value	Accuracy	Sensitivity	Specificity	PPV	NPV
Mortality						
AIMS65	>2	96.30	100	76.62	18.18	100
GBS	>15	77.78	100	96.10	57.10	100
Pre -Rockall	>5	92.59	75	93.51	37.5	98.63
Full Rockall	>6	91.36	75	92.21	33.33	98.61
Intervention required						
AIMS65	>2	56.79	34.88	81.58	68.18	52.54
GBS	>9	61.73	74.42	47.37	61.54	62.07
Pre -Rockall	>6	50.62	6.98	100	100	48.72
Full Rockall	>4	56.79	72.09	39.47	57.41	55.56
Need for ICU						
AIMS65	>1	70.37	72.31	62.5	88.68	35.71
GBS	>10	72.84	69.23	87.50	95.74	41.18
Pre-Rockall	>4	49.38	43.08	75.00	87.50	24.49
Full Rockall	>5	50.62	44.62	75.00	87.88	25.00
Need for blood transfusion						
AIMS65	>0		19.05%	97.44%	88.9%	52.8%
GBS	>9		97.62%	46.15%	66.1%	94.7%
Pre-Rockall	>3		50%	71.79%	65.6%	57.1%
Full Rockall	>4		50%	69.23%	63.6%	56.3%

## Discussion

Various prospective studies have proved the effectiveness of these risk scoring systems in predicting prolonged hospitalization, the requirement for blood transfusion, endoscopic interventions, and mortality [14,15]. International consensus guidelines also stated that early stratification of low from high-risk patients is essential for the management of UGIB with timely interventions to decrease the morbidity as well as mortality burden [16]. However, the available scores have a few limitations; hence comparison between older scores, their simplified versions, and newer risk scores is necessary to direct evidence-based clinical decisions [16, 17]. Hence we compared different risk scoring systems (GBS score, AIMS65 score, and the pre-endoscopy and full Rockall scores) to predict certain clinical outcomes.

Our study had the majority of the subjects (42%) in the 51-65 years age group with a male to female ratio of 3.05:1. In a study done by Chandnani et al., the mean age of the patients was 43.5 years with male predominance (69%) [18]. Thandassery et al. and Nagaraja et al. also reported mean age of 46.16 years in the patients with male predominance [19, 10].

In our study, most patients had liver disease (43.2%) in UGIB and the least common was malignancy (3.7%). Similarly, in Chandnani et al., the study showed liver disease and malignancy in 43.3% and 2.3% of the patients, respectively [19]. The incidence of non-variceal bleed is more frequent, secondary to peptic ulcer disease (41.4%) when compared to variceal bleed (27.9%) [20]. One Chinese study also found that non-variceal UGIB (84.4%) was more commonly observed in patients than variceal UGIB (15.6%) [21]. While our study had comparable variceal and non-variceal bleed cases.

AIMS65 score was better at determining the hospital stay when compared to other risk scoring systems, however not to a statistically significant degree. In our study, patients stayed around 1-15 days in the hospital. However, underlying disease conditions (dyselectrolytemia, regular hemodialysis in chronic kidney disease (CKD) patients in volume overload, sepsis secondary to pneumonia, and urinary tract infections) were the reasons behind longer hospital stay rather than the UGIB.

The AIMS65 score (AUROC: 0.91, cut-off >1, sensitivity 100%, specificity 76.62%) and pre-Rockall were quite similar (AUROC: 0.91, cut-off >0, sensitivity 100%, specificity 93.51%) at predicting mortality than GBS and full Rockall score. Nagaraja et al [10]. stated that the AIMS65 score is better to predict mortality in UGIB patients (AUROC: 0.889) when compared to GBS (AUROC: 0.869). Further, the optimum cut-off for AIMS65 is>2 as compared to >1 in our study [9]. While, Stanley et al. reported GBS better predicts the mortality with AUROC 0.86 (p<0.001) when compared to full Rockall score (0.70), Progetto Nazionale Emorragia digestive (PNED) score (0.69), pre-Rockall score (0.66), and AIMS65 score (0.68) (all P<0.001) [7]. We also found that pre-Rockall and AIMS65 scores had a good NPV of 98.6% and 100 at the cut-offs of >5 and >1 respectively. Hence, scores <1 for AIMS65 and <5 for pre-Rockall has a low risk for mortality in UGIB. Similarly, the study by Saltzman et al. had a similar NPV of 99.7% for a cut-off of >1 [8]. The mortality rate (4.94%) in this study is quite a consensus with the worldwide mortality due to UGIB which is 6-10% [2]; however, less when compared to a study by Chandnani et al (10%) [18]. In this study, 60% of patients who succumbed (3) underwent a blood transfusion, 60% (3) of endoscopic intervention, 80% (4) of them had a variceal bleed, and 20% (1) had a non-variceal bleed. Among the five patients who died, all were males.

We found that both GBS and AIMS65 scores are able to predict ICU requirements in UGIB patients. GBS was the best at predicting the requirement for ICU care (cut-off >9, AUROC 0.793, p<0.001), followed by AIMS65 (cut-off >0, AUROC 0.67, p=0.03). Both scores had a good PPV of 95.7 and 88.7% for GBS and AIMS65, respectively. This helps the clinicians to categorize high from low-risk patients and thus could determine the patients requiring ICU admission. Pines et al in their study also stated both GBS and AIMS65 were able to determine ICU requirements, however, AIMS65 was relatively more accurate compared to GBS [12].

GBS had a higher ability to predict the endoscopic intervention requirement in UGIB patients when compared to other risk scores [7]. Similarly, we also found that GBS was better at predicting the requirement for endoscopic intervention (AUROC 0.618, p 0.06). Chandnani et al. in western India and Tham et al in Glasgow, UK also found that GBS is superior to other risk scores to predict blood transfusion, endoscopic, and radiological or surgical interventions in UGIB [18, 13]. A similar study by Martínez-Cara et al. showed that AIMS65 and GBS were quite identical (0.62 vs. 0.62) in predicting endoscopic intervention and GBS alone was better at predicting blood transfusion requirement [22]. In our study, AIMS65 score cut-off value ≥2 (AUROC .553, p = 0.408, statistically not significant) predicts the blood transfusion requirement. Thandassery et al. in who also had a cut-off value of ≥2 better predicted the blood transfusion requirement (AUROC 0.59) [19]. Our study found that GBS is superior to other risk scores at predicting the requirement for blood transfusion with AUROC being 0.713 with a high sensitivity of 97.62% with the cut-off value of >6, which is also statistically significant (p=0.001).

Our study assessed four pre- and post-endoscopy scores that showed the most promising for clinical use. Hence, we can recognize the optimal way to risk assess UGIB early after the presentation, following endoscopic diagnosis, and following treatment. Comparison of scoring systems might provide invaluable information to the clinicians to keenly identify those who are at high risk of endoscopic intervention and to direct such patients immediately to the intervention. The study has a few limitations that need to be regarded. First, this is a single-center study. Hence, a multi-centric design involving more consecutive patients attending to the hospital needs to be included to validate the current results.

## Conclusions

GBS was superior in predicting categorization into high risk and low risk, and endoscopic intervention, blood transfusion, and ICU care in UGIB patients. Pre-Rockall score and AIMS65 scores were quite comparable in predicting mortality in UGIB patients. GBS and AIMS65 scores help in predicting the requirement for ICU care; AIMS65 being a simple score will also reduce the cost burden of unnecessary ICU admissions.

## References

[1] Ramaekers R, Mukarram M, Smith CA, Thiruganasambandamoorthy V (2016). The predictive value of preendoscopic risk scores to predict adverse outcomes in emergency department patients with upper gastrointestinal bleeding: a systematic review. Acad Emerg Med.

[2] Jain V, Agarwal PN, Singh R, Mishra A, Chugh A, Meena M (2015). Management of upper gastrointestinal bleed. MAMC J Med Sci.

[3] Mahajan P, Chandail VS (2017). Etiological and endoscopic profile of middle aged and elderly patients with upper gastrointestinal bleeding in a tertiary care hospital in north India: a retrospective analysis. J Midlife Health.

[4] Stanley AJ (2012). Update on risk scoring systems for patients with upper gastrointestinal haemorrhage. World J Gastroenterol.

[5] Kim SK, Cho CD, Wojtowycz AR (2008). The ligament of Treitz (the suspensory ligament of the duodenum): anatomic and radiographic correlation. Abdom Imaging.

[6] Mathew P, Kanni PY, Gowda M, Uppalapati S, Garg A, Ansari J (2019). Retrospective study of clinical profile, endoscopic profile and in hospital mortality in acute upper gastrointestinal bleeding in tertiary care centre in south india. Int J Contemp Med Res.

[7] Stanley AJ, Laine L, Dalton HR (2017). Comparison of risk scoring systems for patients presenting with upper gastrointestinal bleeding: international multicentre prospective study. BMJ.

[8] Saltzman JR, Tabak YP, Hyett BH, Sun X, Travis AC, Johannes RS (2011). A simple risk score accurately predicts in-hospital mortality, length of stay, and cost in acute upper GI bleeding. Gastrointest Endosc.

[9] Singh SP, Panigrahi MK (2013). Spectrum of upper gastrointestinal hemorrhage in coastal Odisha. Trop Gastroenterol.

[10] Nagaraja B, Vinay K, Akhila RK, Umesh KJ, Prashant BC (2018). Comparison of prediction of outcomes in upper GI bleed using non-endoscopic scoring systems. Int J Adv Med.

[11] Monteiro S, Gonçalves TC, Magalhães J, Cotter J (2016). Upper gastrointestinal bleeding risk scores: who, when and why?. World J Gastrointest Pathophysiol.

[12] Pines JM, Saltzman JR (2016). Is it time to implement clinical decision rules for upper GI bleeding? Barriers, facilitators, and the need for a collaborative approach. Gastrointest Endosc.

[13] Tham J, Stanley A (2019). Clinical utility of pre-endoscopy risk scores in upper gastrointestinal bleeding. Expert Rev Gastroenterol Hepatol.

[14] Abougergi MS, Charpentier JP, Bethea E (2016). A prospective, multicenter study of the AIMS65 score compared with the Glasgow-Blatchford Score in predicting upper gastrointestinal hemorrhage outcomes. J Clin Gastroenterol.

[15] Robertson M, Majumdar A, Boyapati R (2016). Risk stratification in acute upper GI bleeding: comparison of the AIMS65 score with the Glasgow-Blatchford and Rockall scoring systems. Gastrointest Endosc.

[16] Barkun AN, Bardou M, Kuipers EJ, Sung J, Hunt RH, Martel M, Sinclair P (2010). International consensus recommendations on the management of patients with nonvariceal upper gastrointestinal bleeding. Ann Intern Med.

[17] Cúrdia Gonçalves T, Barbosa M, Xavier S (2018). Optimizing the risk assessment in upper gastrointestinal bleeding: comparison of 5 scores predicting 7 outcomes. GE Port J Gastroenterol.

[18] Chandnani S, Rathi P, Sonthalia N (2019). Comparison of risk scores in upper gastrointestinal bleeding in western India: a prospective analysis. Indian J Gastroenterol.

[19] Thandassery RB, Sharma M, John AK (2015). clinical application of AIMS65 scores to predict outcomes in patients with upper gastrointestinal hemorrhage. Clin Endosc.

[20] Dicu D, Pop F, Ionescu D, Dicu T (2013). Comparison of risk scoring systems in predicting clinical outcome at upper gastrointestinal bleeding patients in an emergency unit. Am J Emerg Med.

[21] Gu L, Xu F, Yuan J (2018). Comparison of AIMS65, Glasgow-Blatchford and Rockall scoring approaches in predicting the risk of in-hospital death among emergency hospitalized patients with upper gastrointestinal bleeding: a retrospective observational study in Nanjing, China. BMC Gastroenterol.

[22] Martínez-Cara JG, Jiménez-Rosales R, Úbeda-Muñoz M, de Hierro ML, de Teresa J, Redondo-Cerezo E (2016). Comparison of AIMS65, Glasgow-Blatchford score, and Rockall score in a European series of patients with upper gastrointestinal bleeding: performance when predicting in-hospital and delayed mortality. United European Gastroenterol J.

